# The Effect of Chicken Extract on Mood, Cognition and Heart Rate Variability

**DOI:** 10.3390/nu7020887

**Published:** 2015-01-29

**Authors:** Hayley Young, David Benton, Neil Carter

**Affiliations:** Department of Psychology, University of Swansea, SA28PP, UK; E-Mails: d.benton@swansea.ac.uk (D.B.); n.carter@swansea.ac.uk (N.C.)

**Keywords:** essence of chicken, heart rate variability, cortisol, stress, mood, cognition

## Abstract

Chicken extract, which is rich in anserine and carnosine, has been widely taken in Asian countries as a traditional remedy with various aims, including attenuation of psychological fatigue. The effects of consuming BRAND’S Essence of Chicken (EOC) or a placebo on 46 young adults’ responses to a standard psychological “stressor” were considered. Heart rate variability (HRV), cortisol responses, mood and cognition were measured at baseline and after ten days supplementation. EOC resulted in feeling less anxious, depressed and confused and more agreeable and clearheaded. A decrease in HRV was observed after EOC but only in females. Cognition and cortisol levels were not influenced by EOC. Findings suggest that EOC may be a promising supplement to improve mood in a healthy population.

## 1. Introduction

Essence of chicken (EOC), a chicken meat extract, in Southeast Asia is traditionally consumed to improve cognition and reduce fatigue. EOC consists mainly of proteins, amino acids and di-peptides such as carnosine, balenine and anserine; peptides that are found in high concentrations in the human brain [1,2]. Previous research has found that drinking EOC for four weeks reduced subjective fatigue after a mental task [3]. Recovery of serum cortisol levels following a stressor was also enhanced in those who consumed EOC for one week [4]. More recently, Konagai *et al.* [5] found that seven days of consuming EOC reduced reaction times, improved working memory and decreased ratings of depression. The general pattern of findings suggests an improved mood and an enhanced ability to recover from a mental workload. The present study sought to replicate and extend these findings by examining the effects of EOC on mood, cortisol and heart rate variability (HRV), both at rest and in response to a cognitive challenge.

Heart rate variability (HRV) is a non-invasive method that monitors autonomic nervous system (ANS) activity and assesses its reactivity under different conditions [6]. Individual differences in HRV, most notably high frequency (HF) power which is thought to reflect parasympathetic activity, have been shown to predict working memory, attention [7] and a person’s ability to regulate emotions [8]. However, little is known about how nutrition affects these processes. Carnosine has been found to inhibit neural activities of sympathetic efferent nerves innervating the adrenal gland and liver, and in rats, facilitates the activity of the vagal celiac nerve that innervates the pancreas [9]. Similarly, after laparotomy an intraduodenal injection of anserine to anaesthetized rats suppressed sympathetic nerve activity and enhanced the activity of the vagal gastric efferent [10]. These mechanisms are thought to mediate the anti-hyperglycaemic effects of carnosine and anserine [11]; however, to our knowledge these effects have not been studied in humans. The primary objective of the present study was to investigate the effects of a supplement containing carnosine and anserine on autonomic nervous system activity in humans using HRV. A secondary objective was to test the effects of EOC on HRV, cortisol levels, cognition and mood.

## 2. Methods

### 2.1. Procedure

At baseline, and after ten days of consumption, participants’ response to a psychological stressor, cognition and mood were assessed. Initially participants were fitted with a heart rate monitor, mood was recorded and a saliva sample (for cortisol measurement) was collected (Section 2.7). Participants then took part in a mildly “stressful task” (Section 2.2) after which mood and cortisol were measured again. Finally, participants completed a cognitive test battery (Section 2.5) and completed a number of questionnaires about their recent mood and general health. A third cortisol sample was collected after the cognitive test battery and a fourth after participants had rested for forty five minutes. Heart rate variability was monitored continually throughout the procedure. At the end of the study participants were paid £60 for their time. The procedure was approved by Swansea University ethics committee No: 07.25.2013.1.

### 2.2. Stress Task

The heart rate response to a standard stressor was examined using the method of Benton *et al.* [12] who found that this procedure increased heart rate by fifteen beats and that a biological intervention, the prior consumption of phosphotidlyserine, ameliorated the increase in heart-rate. Heart rate (HR) was measured using a Polar RS800 HR monitor set to R-R interval mode (Polar Electro, Kempele, Finland), together with an electrode transmitter belt (T61) after the application of a conductive gel. This instrument has been previously validated as accurately measuring R-R intervals and accessing HRV [13,14].

Stage one—Initial relaxation (5 min). Initially mood was reported and then HR was monitored for five minutes, in a dimly lit room, while listening to calming music (Tranquility of Baroque, Warner Music). Each interbeat interval was recorded but to allow a sufficient window length for HRV analysis data are reported as an average over five minute periods.

Stage two—Stressor (5 min). Mental arithmetic was then performed for five minutes. A tape-recorder presented a series of additions and subtractions involving double-digit numbers. A tone sounded after two seconds at which time the subject was expected to respond. Average HRV over the period was calculated. The speed of presentation was designed to be just too quick to allow consistently accurate answers.

Stage three—Recovery (5 min). The decline in heart rate was then recorded for a further five minute period while the subjects sat quietly. Mood was measured for a second time.

### 2.3. Participants

Forty six undergraduates (Table 1) gave informed consent, and under a double-blind procedure were randomly allocated to one of two groups who did or did not consume EOC. The sample size is based on the positive results of Benton *et al.* [12] who used the same “stress” paradigm (Section 2.2).

**Table 1:** Descriptive data for participants who consumed EOC/placebo.

		EOC	PLACEBO	*p*
Gender	Female	13	11	n.s.
	Male	11	11	n.s.
Age		22.2 (3.6)	21.5 (2.7)	n.s.
BMI		23.4 (4.5)	23.2 (2.9)	n.s.

### 2.4. Nutritional Intervention

Participants consumed 1 bottle (70 mL) of either EOC or a placebo for ten days. A bottle of EOC (70 mL) contains 83 mg protein and peptide, 3.1 mg free amino acids, 0.8 mg hexose, and 0.4 mg fat [15]. It also contains β-alanyl-l-histidine (carnosine) and β-alanyl-l-methyl-l-histidine (anserine) as active di-peptides [15]. The dose of EOC used in the present study was based on previous human studies [3,16,17]. A bottle of placebo (70 mL) contained 83 mg milk casein and 3mg caramel to produce a protein content, caloric content, and colour similar to EOC. Casein was chosen as an ingredient in the placebo because it does not include peptides reported to have effects on fatigue and has a similar amino-acid composition as EOC. The EOC and placebo samples were provided by Cerebos Pacific Ltd. (Singapore).

### 2.5. Cognitive Test Battery

#### 2.5.1. Working Memory—Serial Sevens

A computerized version of the serial sevens task was used in which subjects were required, from a starting number between 800 and 999, to say whether a second number was exactly seven less. The test was scored as the total number of correct subtractions and the average of the time taken, in milliseconds, to perform each subtraction.

#### 2.5.2. Selective Attention—Arrow Flankers Test

A modified version of the Eriksen and Eriksen [18] flanker task was used to measure selective attention. The Arrow Flankers test measures the ability to direct attention and ignore peripheral information. The flanking pairs of symbols could be squares (□□ < □□), crosses (xx < xx), congruent arrows (pointing in the same direction (>> > >>)) or incongruent arrows (pointing in the opposite direction (>> < >>)). A stimulus remained on screen until the key press was registered and there was a randomly varying inter-stimulus interval of between 1 and 3 s. The task was to indicate whether the middle arrow is pointing to the right or left and the reaction times (in milliseconds) and accuracy (number incorrect) were recorded.

#### 2.5.3. Simple and Choice Reaction Times

The reaction time procedure was based on that of Jensen [19]. On a panel eight lamps were arranged in a semicircle 5.5 inches from a central button (the home key). The index finger was placed on the home key. Within one to two seconds an auditory warning signal sounded and after a random interval of one to four seconds one of the lamps illuminated. The subject then extinguished the light by depressing a button directly below the lamp, using the finger initially on the home key. All subjects completed a practice session of 20 trials utilising all eight lamps. Simple reaction times were measured for 20 trials using one lamp. Choice reaction times were then measured over three sets of 20 trials when one of 2, 4 or 8 lamps could be potentially illuminated. Decision times, the time taken to lift the finger from the home key, and movement times, the time from the hand leaving the home key to pressing the button under the illuminated light, were analysed.

### 2.6. Mood

#### 2.6.1. Profile of Mood States

The Profile of Mood States Bi-Polar Form (POMS) [20] is a 72-item self-report questionnaire that measures six dimensions of mood: (1) Composed—Anxious; (2) Energetic—Tired; (3) Elated—Depressed; (4) Clear-headed—Confused; (5) Agreeable—Hostile; (6) Confident-Unsure. Participants were presented with a list of words or phrases and had to rate on a scale of 0–3 (0 “not at all”; 3 “a lot like this”) how much they had felt like this in the past week including today. There are twelve words for each mood dimension—six positive and six negative.

#### 2.6.2. Visual Analogue Scales

Changes in mood during the test battery were assessed using 100 mm visual analogue scales that correspond to the dimension of the POMS: Anxious; Hostile/Agreeable; Elated/Depressed; Unsure/Confident; Energetic/Tired; Confused/Clearheaded. Participants were asked to report how they felt “at this moment” by placing a cross on each of the scales. Mood (VAS) was measured prior to the stressful task, immediately following the stressful task and again after completing the cognitive test battery.

#### 2.6.3. General Health Questionnaire (GHQ)

The GHQ is a 30-item self-report questionnaire that was developed to detect in a community sample those who would benefit from seeing a psychiatrist [21]. The participant responds to various statements concerning mental health by rating themselves on a four point scale that ranges from “better/healthier than normal”; “same as usual”; “worse/more than usual” to “much worse/more than usual”. These are scored using a scale 0-1-2-3, as the responses vary from positive to negative, such that a higher score indicates a greater incidence of psychiatric problems. Based on a factor analysis by Chan [22] the GHQ was divided into subscales representing Depression, Anxiety, Social functioning and Quality of Sleep.

#### 2.6.4. Perceived Stress Scale

The Perceived Stress Scale [23] is a 10-item self-report questionnaire that assesses the degree to which situations in one’s life are perceived as stressful. The participants were required to answer questions about the extent to which they have had stressful thoughts and feeling during the last week, for example, “In the last week, how often have you been upset because of something that happened unexpectedly?” The participant responds on a 5-point scale (ranging from 0 = Never to 4 = Very Often). One overall score was produced by summing all items.

### 2.7. Cortisol

Before and after completing the stressful task, after completing the test battery and again after a 45 min rest to monitor recovery, a sample of saliva was collected and used to assay cortisol levels. Testing was carried out in the afternoon when cortisol levels have reached a plateau. Analysis was carried out using an immunoassay supplied by Salimetrics Europe Ltd., Unit Newmarket, Suffolk, UK.

### 2.8. Heart Rate Variability

HRV data were transferred to Polar Pro Trainer 5 software (Polar Electro, Kempel, Finland) and each downloaded R-R interval file was then further analysed using Kubios HRV Analysis Software 2.0 (The Biomedical Signal and Medical Imaging Analysis Group, Department of Applied Physics, University of Kuopio, Finland) [24]. Data were manually inspected for artefacts caused by ectopic beats, poor conductivity, *etc.* A very low correction threshold was chosen for artefact correction (0.45 from local average) so as not to distort natural variability. Less than 1% of beat were identifies as artefacts, however, two cases were removed based on very poor recording and two were removed based on abnormal HRV responses. The latter cases were considered extreme outliers that without removal would have significantly altered the final analysis.

Time domain HRV indices included average interbeat interval (R-R) (a measure of basic heart rate), standard deviation of interbeat interval (SDNN) (measures total variability in the sample) and the root mean squared of the successive differences (rMSSD) (higher values indicate stronger vagal activity).

Spectral analysis was conducted to transform the time series into the frequency domain. The R-R interval series was converted to equidistantly sampled series by cubic spline interpolation at a rate of 4 Hz. Welsh’s periodogram, which divides the R-R series into overlapping windows, was used to decrease the leakage effect, and the spectrum estimate was obtained by averaging the Fast Fourier Transform (FFT) spectra of these windowed segments. Estimates of Low frequency (LF) (0.04–0.15 Hz) and High frequency (HF) (0.15–0.4), which represent the influence of sympathetic and parasympathetic activity respectively were obtained. The ratio of LF to HF (LF/HF) which represents overall autonomic nervous system balance was also considered. All procedures were conducted in accordance with the recommendations of Task Force of the European Society of Cardiology and The North American Society of Pacing and Electrophysiology [25].

### 2.9. Statistical Analysis

The data were examined using appropriate analysis of co-variance designs with performance on visit 2 as the dependant variable and performance on visit 1 as the covariate. Where there are repeated measures on visit 1 the first measure (baseline) was used as the covariant. EOC/placebo and gender were entered as between subject factors. Where applicable additional repeated measures factors were also included in the model, for example with mood, time (before/after testing) was entered. Where predicted interactions were significant, planned comparisons were calculated to determine the nature of the interaction. Unless otherwise stated the effect of the covariant was significant. The data reported are adjusted means (SE).

## 3. Results

Table 1 reports the descriptive data for participants who drank either the EOC or the placebo drinks. The original sample consisted of 50 subjects of which 46 (92%) completed the study. Of the four subjects that did not return for their second visit, three were allocated to the placebo and one to the EOC condition (Chi2 (1, *n* = 50) = 1.087 n.s.). To assess compliance to the experimental protocol subjects were also asked how many drinks they actually consumed; they were informed that this would not affect their payment. 65.2% (*n* = 30) reported consuming all ten drinks as instructed; the number of subjects in each condition who reported consuming all ten drinks was equal (EOC *n* = 15, Placebo *n* = 15). Of those that did not consume all ten drinks, 21.7% (EOC *n* = 5, Placebo *n* = 5) consumed nine drinks, 8.7% (EOC *n* = 2, Placebo *n* = 2) consumed 8 drinks and 4.3% (EOC *n* = 2, Placebo *n* = 0) drank seven of the drinks. Therefore the number of drinks consumed did not depend on the condition to which the participants had been allocated to (Chi2 (3, *n* = 46) = 1.087, n.s.). When asked if they thought they were taking an active ingredient, a placebo or don’t know, 19.6% (EOC *n* = 2, Placebo *n* = 7) said they were taking an active substance, 23.9% (EOC *n* = 7, Placebo *n* = 4) thought they took the placebo and 56.5% (EOC *n* = 15, Placebo *n* = 11) were unsure. The drink participants thought they consumed did not depend on the condition to which they had been allocated to (Chi2 (2, *n* = 46) = 4.132, n.s.); therefore the blind was successful. When asked about any side effects 84.8% (EOC *n* = 20, Placebo *n* = 19) reported no side effects, one person, who took the placebo, reported an inability to concentrate and another reported sleeplessness. Of those taking the EOC treatment, one reported feeling more thirsty than usual, one reported decreased bowel movements, one reported feeling more tired and another thought they experienced slight insomnia. Again none of these effects depended upon which drink had been consumed (Chi2 (6, *n* = 46) = 6.091, n.s.).

Baseline data are presented in Table 2. At baseline participants who subsequently consumed the placebo were more clearheaded than those who consumed EOC. There were no other significant differences at baseline between those who subsequently consumed EOC or a placebo.

**Table 2:** Baseline data (mean (SE)) for participants who consumed EOC/Placebo.

		EOC	PLACEBO
Decision times		192.4 (9.9)	172.9 (10.3)
Movement times		365.1 (12.6)	337.4 (13.1)
Serial sevens RT		1895.9 (161.3)	2033.3 (211.0)
Serial sevens errors		2.6 (0.4)	1.8 (0.5)
Focused attention RT	Congruent	567.5 (14.2)	560.8 (14.8)
	Neutral	574.9 (16.3)	556.1 (17.4)
	Incongruent	630.3 (16.2)	626.9 (16.9)
Focused attention errors	Congruent	0.84 (0.1)	0.2 (0.1)
	Neutral	1.0 (0.6)	0.1 (0.6)
	Incongruent	0.9 (0.2)	0.7 (0.2)
Perceived stress		17.8 (1.3)	17.3 (1.4)
General health questionnaire	Anxiety	7.1 (0.8)	7.8 (0.9)
	Sleep	2.6 (0.3)	2.4 (0.3)
	Depression	5.0 (0.6)	4.2 (0.6)
	Social functioning	5.1 (0.4)	5.6 (0.4)
	Total GHQ	28.4 (2.4)	30.2 (2.6)
POMS	Depressed/Elated	4.5 (1.2)	4.1 (1.3)
	Anxious/Composed	0.3 (1.3)	0.5 (1.4)
	Agreeable/Hostile	7.9 (1.0)	7.6 (1.1)
	Confident/Unsure	1.6 (1.3)	0.5 (1.4)
	Energetic/tired	−2.0 (1.2)	−3.0 (1.3)
	Clearheaded/Confused	1.6 (1.2)	3.3 (1.4)
VAS	Depressed/Elated	217.8 (8.5)	219.3 (8.9)
	Anxious/Composed	221.5 (9.9)	243.1 (10.2)
	Agreeable/Hostile	255.4 (7.5)	260.9 (7.8)
	Confident/Unsure	212.0 (9.4)	232.5 (9.8)
	Energetic/Tired	187.3 (9.4)	179.4 (9.6)
	Clearheaded/Confused	222.8 (8.2) **	250.5 (8.6) **
HRV	R-R	761.9 (19.7)	803.9 (20.5)
	SDNN	68.3 (5.1)	70.4 (5.2)
	rMSSD	47.8 (5.3)	50.0 (6.8)
	LF power	1464.2 (230.5)	1454.3 (263.6)
	HF power	960.69 (160.3)	961.4 (166.3)
	LF/HF	2.1 (0.3)	1.9 (0.2)
Cortisol		0.21 (0.02)	0.20 (0.02)

** *p* < 0.01; Cohen’s *d* = 0.6.

### 3.1. Heart Rate Variability

The mean R-R interval was calculated over three five minute periods (Rest: prior to “stress” task; Active: during the task; Recovery: after the task). For each of the HRV indices data were analysed using a three way ANCOVA (Time × Gender × Variant (EOC or placebo). For each HRV index its respective “rest” measure on the first visit was used as the covariant.

#### 3.1.1. Time—Domain Analysis

The interaction Time (Rest/Active/Recovery) × Variant (EOC/Placebo) was not significant (F(2,72) = 1.453, n.s.). However, there was a main effect of time (F(2,72) = 3.484, *p* < 0.03). The R-R interval during the task was shorter (Rest: 787.9 (15.7); Active: 735.3 (12.2); Recovery: 801.4 (15.0)). This effect shows that the “stressor” did, as planned, lead to an increase in heart rate and as such the participants were suitably “stressed” by the task. However, consuming EOC did not affect heart rate during the task. When SDNN was considered the interaction Time × Variant was again non-significant (F(2,74) = 0.660, n.s.). The main effect of time was significant (F(2,74) = 4.882, *p* < 0.01). SDNN reduced during the task (Rest: 67.2 (5.2); Active: 66.9 (4.6); Recovery: 77.0 (4.9). Again this effect shows that the “stressor” was effective. Similarly when rMSSD was considered the interaction Time × Variant was not significant (F(2,74) = 0.983, n.s.). The main effect of time was significant (F(2,74) = 4.058, *p* < 0.02). rMSSD reduced during the task (Rest: 45.9 (6.4); Active: 40.8 (4.5); Recovery: 48.9 (6.1) suggesting that the task was effective although EOC did not influence rMSSD. There were no effects involving gender for any of the time domain HRV indices.

#### 3.1.2. Frequency—Domain Analysis

LF and HF power and their ratio were calculated over three five minute periods. When the influence of consuming EOC on LF power were considered there was no effect (Time × Variant (F(2,72) = 0.40, n.s.). Similarly, there were no effects of time or gender. When HF power was considered all effects were again non-significant; (Time × Variant (F(2,72) = 0.10, n.s.) Again there were no effects involving time or gender.

**Figure 1:**
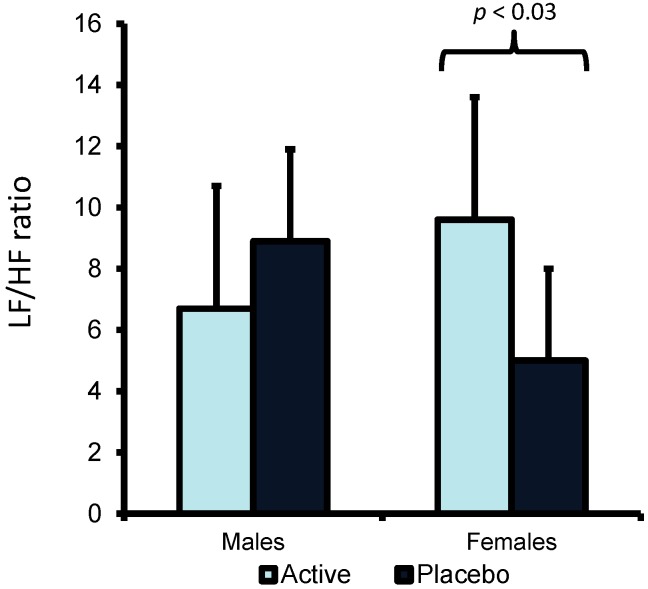
The effect of Essence of Chicken (EOC) on LF/HF ratio in males and females. Data are mean (SE) for Low Frequency/High Frequency (LF/HF) ratio. Females but not males, who drunk EOC rather than placebo, had a higher LF/HF ratio (*p* < 0.03).

However, with the LF/HF ratio there was a Variant X Gender interaction (F(1,36) = 8.944, *p* < 0.005). Follow up tests revealed that females but not males, who had drunk EOC rather than placebo, had a higher LF/HF ratio (Figure 1).

### 3.2. Cortisol

Cortisol was measured when participants arrived at the laboratory (Time 1), immediately following the difficult task (Time 2), again after the cognitive battery (Time 3) and after a 45 min recovery period (Time 4). Data were considered using a three way ANCOVA (Time (1, 2, 3, and 4) × Gender × Variant (EOC/Placebo)). Baseline (Time 1) cortisol on the first visit was used as the covariate. The interaction Variant × Time (Time 1, 2, 3, 4) was not significant (F(3,123) = 0.908, n.s.). However, there was a main effect of time (F(3,123) = 13.344, *p* < 0.001); participants cortisol levels were highest when they first arrived at the laboratory (Time 1) and steadily declined until the end of the procedure (Time 4). This finding suggests that the “stressor” did not activate the hypothalamic-pituitary-adrenal axis. To replicate the findings of Nagai *et al.* [4] change scores were calculated (Time 4 minus Time 3) to represent recovery from immediately after the test battery to forty five minutes later. There was a trend for those who consumed EOC to recover more quickly (−0.05 for EOC, −0.03 for Placebo) but this effect did not reach significance (F(1,42) = 1.969, *p* < 0.1). There were no effects of gender.

### 3.3. Serial Sevens

#### 3.3.1. Reaction Times

The interaction Variant (EOC/Placebo) × Gender was non-significant (F(1,42) = 0.020, n.s.), and neither was the main effect of variant (F(1,41) = 2.519, n.s.).

#### 3.3.2. Accuracy

The interaction Variant (EOC/Placebo) × Gender was non-significant (F(1,42) = 1.795, n.s.) and neither was the main effect of variant (F(1,42) = 1.695, n.s.).

### 3.4. Focused Attention—Arrow Flankers

#### 3.4.1. Congruent Stimuli

When reaction times were considered the interaction Variant (EOC/Placebo) × Gender was non-significant (F(1,41) = 0.162, n.s.) as were all other interactions. Similarly, when the number of incorrect responses were considered all effects were non-significant; Variant (EOC/Placebo) × Gender interaction was non-significant (F(1,41) = 2.468, n.s.).

#### 3.4.2. Neutral Stimuli

When reaction times were considered the interaction Variant (EOC/Placebo) × Gender was non-significant (F(1,40) = 0.001, n.s.) as were all other interactions. With the number of incorrect responses, all effects were non-significant; Variant (EOC/Placebo) × Gender (F(1,40) = 0.098, n.s.).

#### 3.4.3. Incongruent Stimuli

When reaction times were considered the interaction Variant (EOC/Placebo) × Gender was non-significant (F(1,41) = 0.789, n.s.) as were all other interactions. With the number of incorrect responses, all effects were non-significant; Variant (EOC/Placebo) × Gender (F(1,41) = 0.774, n.s.).

### 3.5. Reaction Times

#### 3.5.1. Movement Times

Neither the Variant (EOC/Placebo) × Gender × Lamps (1, 2, 4, 8 lamps) (F(3,123) = 0.027, n.s.) nor any other interactions or main effects reached significance.

#### 3.5.2. Decision Times

The interaction Variant (EOC/Placebo) × Gender × Lamps (1, 2, 4, 8 lamps) was non-significant (F(3,123) = 0.382, n.s.), however, the interaction Variant (EOC/Placebo) × Lamps (1, 2, 4, 8 lamps) reached significance (F(1,123) = 3.477, *p* < 0.01). Those who consumed EOC tended to have faster decision time on the 8 lamp task (174.7 (6.6) for EOC; 199.5 (6.9) for Placebo) and the 4 lamp task (167.4 (5.6) for EOC; 187.9 (5.8) for Placebo) but follow up tests did not reach significance.

### 3.6. Mood (VAS)

To assess the effects of treatment on mood and changes in the response to testing, a 3-way ANCOVA was conducted for each VAS scale: Variant (EOC/Placebo) × Gender × Time (0, 30, 60 min) with the initial (baseline) rating on day 1 as the covariant.

#### 3.6.1. Energetic/Tired

The Variant (EOC/Placebo) × Gender × Time (0, 30, 60 min) was non-significant (F(2,86) = 0.063, n.s.), as was the main effect of Variant (F(1,41) = 0.130, n.s.) The interaction Variant × Time approached significance (F(2,86) = 2.684, *p* < 0.07); participants tended to be more energetic at the end of testing if they drunk EOC but as this effect did not reach significance it should be interpreted with caution.

#### 3.6.2. Agreeable/Hostile

**Figure 2:**
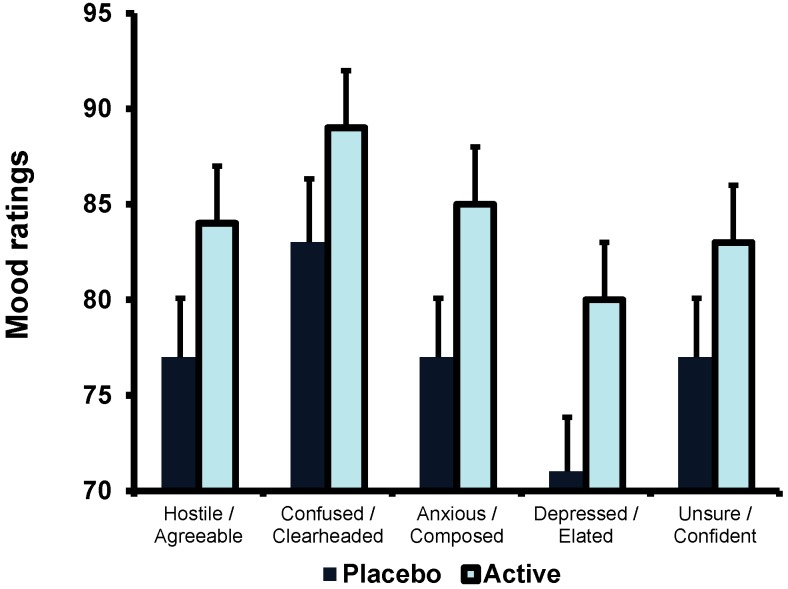
The effect of EOC on ratings of mood. Data are mean (SE) for the sum of all three time points on visit 2 for ratings of agreeableness, clear-headedness, anxiety, depression and confidence. Participants who had drunk EOC, rather than placebo, rated themselves more agreeable (*p* < 0.007), more clearheaded (*p* < 0.05), less anxious (*p* < 0.04), less depressed (*p* < 0.01) and more confident (*p* < 0.05).

The Variant (EOC/Placebo) × Gender × Time (0, 30, 60 min) was not significant (F(2,82) = 0.380, n.s.), however, the main effect of Variant (EOC/Placebo) reached significance (F(1,41) = 8.190, *p* < 0.007; Figure 2). Participants who consumed EOC rated themselves as more agreeable than those who had drunk the placebo (89.3 (1.4) for EOC, 83.4 (1.4) for Placebo). As there was no effect of time neither the “stressful” task nor the cognitive test battery affected participants’ ratings of agreeableness. There was no effect of Gender.

#### 3.6.3. Clearheaded/Confused

The Variant (EOC/Placebo) × Gender × Time (0, 30, 60 min) was not significant (F(2,82) = 0.515, n.s.), however, there was a main effect of Variant (EOC/Placebo) (F(1,43) = 3.808, *p* < 0.05; Figure 2). Participants who consumed EOC were significantly more clearheaded compared to those that consumed the placebo (85.0 (2.5) for EOC, 77.5 (2.7) for Placebo). There was also a main effect of gender (F(1,42) = 11.378, *p* < 0.002); males reported feeling more clearheaded than females (Males: 85.8 (2.4); Females 74.6 (2.3)). As the effect of time was not significant completing the tasks did not influence participants’ ratings of confusion.

#### 3.6.4. Composed/Anxious

The Variant (EOC/Placebo) × Gender × Time (0, 30, 60 min) was non-significant (F(2,80) = 353, n.s.), however, there was a main effect of Variant (EOC/Placebo) (F(1,40) = 3484, *p* < 0.04; Figure 2). Participants who consumed EOC were significantly less anxious than those who had drunk the placebo (84.9 (2.4) for EOC; 77.4 (2.5) for Placebo). Gender did not affect anxiety ratings and the effect of time was not significant.

#### 3.6.5. Elated/Depressed

The Variant (EOC/Placebo) × Gender × Time (0, 30, 60 min) was non-significant (F(2,82) = 0.786, n.s.), however, there was a main effect of Variant (EOC/Placebo) (F(1,41) = 6.544, *p* < 0.01; Figure 2). Those who consumed the placebo rated themselves as more depressed than those who consumed EOC (79.8 (2.3) for EOC, 71.0 (2.4) for Placebo). Neither gender nor time influenced the ratings of depression.

#### 3.6.6. Confident/Unsure

The Variant (EOC/Placebo) × Gender × Time (0, 30, 60 min) interaction was non-significant (F(2,82) = 0.075, n.s.), however, there was a main effect of Variant (EOC/Placebo) (F(1,41) = 3.987, *p* < 0.05; Figure 2). Subjects who consumed EOC were significantly more confident compared to who had drunk the placebo (83.0 (2.0) for EOC; 77.1 (2.1) for Placebo). There was no effect of Gender. In addition, as there was no effect of time, neither the “stressful” task nor the cognitive test battery affected confidence ratings.

### 3.7. Profile of Mood States

When ratings on the POMS were considered the findings were similar to those for the VAS. Participants were more confident (F(1,40) = 3.896, *p* < 0.05) and less depressed (F(1,39) = 5.639, *p* < 0.02) if they had drunk EOC. However, no effects of EOC on anxious/calm, clearheaded/confused, agreeable/hostile, or energetic/tired were observed.

### 3.8. Perceived Stress Scale

The interaction Variant (EOC/Placebo) × Gender was not significant (F(1,40) = 0.028, n.s.) and neither was the main effect of Variant (F(1,40) = 0.872, n.s.).

### 3.9. General Health Questionnaire

Neither the interaction Variant (EOC/Placebo) × Gender (F(1,38) = 0.002, n.s.) nor the main effect of variant (F(1,38) = 0.958, n.s.) were significant. Similarly, there were no effects of treatment on any of the individual subscales; Anxiety, Quality of Sleep, Depression and Social functioning.

## 4. Discussion

The present study aimed to examine the influence of EOC on mood in general and also as a response to a challenging cognitive test battery. It was hypothesised that consuming EOC would increase energy levels and reduce ratings of anxiety and depression. Previous studies have found that taking EOC was associated with increased energy; a recent study by Yamano *et al.* [3] examined the effects of 28 days supplementation of EOC on daily ratings of fatigue and found that when participants consumed EOC, rather than a placebo, they rated themselves as less fatigued. Similarly, Nagai *et al.* [4] found that subjects’ energy levels declined less during a mental arithmetic if they drank EOC rather than a placebo. Although there was a trend towards increased energy levels after EOC in the present (*p* < 0.07) study the effect did not reach significance. It is, however, possible that with a larger sample size this effect would have been significant supporting the findings of Yamano *et al.* [3] and Nagai *et al.* [4]. Also consistent with a beneficial effect of EOC on energy levels was the increased LF/HF ratio observed in the present study (Figure 1). This may indicate that consuming EOC is associated with a general increase in arousal which may be perceived as producing greater levels of energy.

In support of our hypothesis, the present study found that participants who had drunk EOC reported feeling less depressed and anxious and more confident, clearheaded and agreeable (Figure 2). Azhar *et al.* [16] reported that participants, who had drunk EOC, reported fewer mild psychiatric symptoms (GHQ) after two weeks. Similarly a measure of the quality of life improved (SF36 Health Status Survey) in those consuming EOC. However, we found no evidence of an association between EOC and total GHQ ratings or any of its subscales; Anxiety, Quality of Sleep, Depression and Social functioning. It is, however, interesting that in our study participants who consumed EOC rated themselves as less depressed and less anxious on both versions of the POMS (72 item questionnaire and VAS), but that these effects were not detectable on the GHQ depression or anxiety subscales. The GHQ was developed to detect in a community sample those who would benefit from seeing a psychiatrist [21] and our sample scored very low on the depression (4.7 (3.1)/21) and anxiety (7.3 (4.5)/21) subscales. It is possible that a questionnaire designed to distinguish those who may and may not have an increased chance of depression may not be sensitive enough to detect short-term differences in well-functioning young adults. The average rating on the depression VAS was 73.2 (17.6) and on the anxiety VAS was 77.8 (21.8), suggesting that our sample was generally calm and happy, therefore, supplementing with EOC may improve mood in a non-depressed or anxious population, however, research is needed to establish whether there are any effects of EOC on mood in a clinical sample.

A further aim of the present study was to examine whether consuming EOC influences autonomic nervous system activity at rest and in response to a stressful task. Based on animal research, which found anserine and carnosine reduced sympathetic nervous system activity [9], it was hypothesised that EOC would produce the same effects in humans. To our knowledge only one study has previously considered the effects of EOC on autonomic nervous system activity. Shin and Moritani [26] compared the acute effects (1.5 h after a single dose) of consuming either EOC, a combination of capsaicin, green tea extract and EOC, or a placebo, on indices of autonomic nervous system activity (very low frequency (VLF), LF, HF and total power) in six healthy males. They found that the combination of capsaicin, green tea extract and EOC increased VLF, LF and total power but EOC taken alone had no effect [26]. The present findings are consistent with those of Shin and Moritani [26]; there was no evidence for an effect of EOC on indices of autonomic nervous system activity in males. However females who consumed EOC had a higher LF/HF ratio compared to those who consumed the placebo (Figure 1). The finding would suggest either an increase in sympathetic nervous system activity or a decrease in vagal activity after drinking EOC. This is in contrast to animal research which suggested that carnosine/anserine reduced sympathetic and increased parasympathetic nervous system activity [9,10]. However, this research was conducted using anaesthetised rats and it is possible that different results would have occurred when awake. Gender differences in cardiovascular reactivity have been frequently documented [27,28] which may explain why consuming EOC only influenced HRV in females. Although there was a significant effect of EOC on HRV it did not influence the participants’ response to a mental challenge: rather there was a general increase in HRV regardless of whether subjects were active or at rest. Given that HRV is related to mood and in particular depression [29] it is possible that the effect of EOC on mood may be related to changes in HRV. Further research is needed to elucidate in humans further the effects of EOC on HRV indices.

As a further index of subjects’ stress levels we measured salivary cortisol at baseline, in response to the tasks and after a recovery period. It has been previously reported that after a mental workload the speed of recovery of serum cortisol levels was enhanced in those who had consumed EOC [4]. Given that elevated cortisol levels are associated with poorer cognition [30] and mood [31], the enhanced cortisol recovery may partially explain the beneficial effects of EOC on cognition and mood. However, the present study was unable to replicate these findings possibly due to the smaller sample size. In addition, we did not find an increase in cortisol in response to the challenging task; rather participants’ cortisol was highest upon arrival and steadily declined thereafter. This is perhaps not surprising given the half-life of cortisol is about one hour and our challenging task latest only five minutes. Nonetheless if EOC were to influence hypothalamic-pituitary axis (HPA) activity we might have observed a significant main effect of EOC but we did not. It should also be considered that the “stressful task” used in the present study was designed to be mild; that is to represent daily challenges. These findings may not generalise to more severe stressors that have a more profound influence on mood and cortisol responses, for example, the “Trier social stress test”. Therefore, the present study does not support a beneficial effect of EOC on HPA activity in general but further research is required to determine the effect of EOC on HPA activity in response to more prolonged “stressors”.

Previous research has also suggested that EOC may improve aspects of cognition, in particular working memory [4,16] and attention [3,5]. Unfortunately, the present study was unable to replicate these findings; drinking EOC was not associated with any aspect cognitive performance. A possible explanation for this is the smaller sample size that was used in the present study; nonetheless more research is needed to elucidate the effects of EOC.

Since EOC contains many different components it is difficult to identify the active ingredient that may enhance mood; EOC consists mainly of proteins, peptides and free amino acids [15]. Among the peptides carnosine and anserine are also present at relatively high concentrations in the human brain [2] and may contribute toward neuronal protection [32], possibly via their antioxidant [2,33] or antiglycation [34] activities. For example, rats supplemented with a chicken breast extract or carnosine showed significantly reduced depression like symptoms (measured by the forced swimming test) and had decreased 3-methoxy-4-hydroxyphenylglycol (a major metabolite of norepinephrine) levels in their hippocampus [35]. Furthermore, supplementing chicken extract has been shown to increase carnosine and anserine levels in animals’ brains [36]. This suggests that the anti-depressant like effects of chicken extract effect may be due, in part, to its major components, carnosine and anserine.

The mechanisms mediating the beneficial effects EOC or carnosine are not, however, understood. It is possible that EOC, through virtue of its histidine containing peptides, may exert its effects via the histaminergic system in the brain [15]. Histidine is the precursor to histamine and therefore has an important role in the maintenance of wakefulness [37], possibly reducing fatigue. There is also some evidence that consuming EOC may modulate cerebral levels of 5-HT. Xu and Sim [38] found that EOC markedly increased the level of 5-hydroxyindoleacetic acid (5-HIAA) in the cerebrospinal fluid, the main metabolite of 5-HT, a finding consistent with our data showing reduced feelings of depression and anxiety. Recently, Tsuruoka *et al.* [39] isolated a Diketopiperazine (cyclo(l-Phe-l-Phe)) from EOC and found that it was a serotonin transport inhibitor that increased cerebral monoamine levels and significantly improved depressive behaviour in mice. These findings suggest that the benefits of EOC on mood may be mediated by serotonergic mechanisms although more research is needed to confirm this hypothesis.

There remain many unanswered questions: further research is needed to establish whether the beneficial effects of EOC are observed after a single acute dose or whether longer term dietary supplementation is required. In the studies to date EOC has been consumed for between seven [4] and twenty eight days [3] with participants returning for testing on the last day of supplementation. Given that subjects will have taken EOC only hours before testing, the use of this approach makes it difficult to disentangle acute from longer term benefits. It should also be considered that the large number of statistical tests carried out in the present study may have raised the possibility of type 1 errors. However, the present findings are in line with previously observed benefits of EOC, in particular its effect on mood [2,3]. Nonetheless, the present findings should be regarded as exploratory and are in need of replication.

## 5. Conclusions

In conclusion, the present studies found that consuming EOC daily for ten days improved mood in males and females and increased LF/HF HRV in females. Future research should focus on isolating the active ingredients of EOC and the mechanisms mediating its effects, including possible effects on autonomic nervous system activity.
